# Meniscal substitution, a developing and long-awaited demand

**DOI:** 10.1186/s40634-020-00270-6

**Published:** 2020-07-25

**Authors:** Philipp W. Winkler, Benjamin B. Rothrauff, Rafael A. Buerba, Neha Shah, Stefano Zaffagnini, Peter Alexander, Volker Musahl

**Affiliations:** 1 Department of Orthopaedic Surgery, UPMC Freddie Fu Sports Medicine Center, University of Pittsburgh, 3200 S. Water St, Pittsburgh, PA 15203 USA; 2 Department for Orthopaedic Sports Medicine, Klinikum rechts der Isar, Technical University of Munich, Ismaninger Str. 22, 81675 Munich, Germany; 3 Center for Cellular and Molecular Engineering, University of Pittsburgh, 450 Technology Drive, Suite 239, Pittsburgh, PA 15219 USA; 4 2° Clinica Ortopedica e Traumatologica, Istituto Ortopedico Rizzoli, IRCCS, University of Bologna, Bologna, Italy

**Keywords:** Knee, Meniscus, Allograft, Transplantation, Scaffold, Tissue engineering, Substitute

## Abstract

The menisci represent indispensable intraarticular components of a well-functioning knee joint. Sports activities, traumatic incidents, or simply degenerative conditions can cause meniscal injuries, which often require surgical intervention. Efforts in biomechanical and clinical research have led to the recommendation of a meniscus-preserving rather than a meniscus-resecting treatment approach. Nevertheless, partial or even total meniscal resection is sometimes inevitable. In such circumstances, techniques of meniscal substitution are required. Autologous, allogenic, and artificial meniscal substitutes are available which have evolved in recent years. Basic anatomical and biomechanical knowledge, clinical application, radiological and clinical outcomes as well as future perspectives of meniscal substitutes are presented in this article. A comprehensive knowledge of the different approaches to meniscal substitution is required in order to integrate these evolving techniques in daily clinical practice to prevent the devastating effects of lost meniscal tissue.

## Background

The knee is the most commonly injured part of the body among young athletes [1], with isolated meniscal injuries occurring in approximately 15% of acute knee traumas associated with hemarthrosis [2]. The incidence of acute meniscal tears in combined ligamentous injuries is even higher, ranging up to 82% [3]. Differing in etiology and pathogenesis, degenerative meniscal injuries more often affect the elderly population and increase with age, with an estimated prevalence of over 50% between 70 and 90 years of age [4, 5].

Intensive research efforts have established a comprehensive knowledge of the menisci and their essential role for long-term knee function. This is reflected in the decreasing number of meniscal resections and the increasing number of meniscal repairs, highlighting a trend towards an evidence-based and meniscus-preserving approach in the treatment of meniscal tears. Among U.S. orthopedic surgeons, a decrease of 17% and an increase of 37% for meniscectomy and meniscal repair cases per surgeon, respectively, was observed from 2004 to 2012 [6]. Nevertheless, depending on the meniscal tear pattern, the tear size, location, quality of the meniscal tissue, and the stability of the meniscal tear and the knee joint, some meniscal tears are not reparable. In a recent study, 65% of 2066 meniscal tears were considered as irreparable [7]. Thus, a partial or total meniscectomy sometimes remains the best or only treatment option, although the devastating effects and radiographic changes of meniscectomy have been established [8]. Several studies have remarkably demonstrated the increased rate of knee osteoarthritis (OA), requirement for total knee arthroplasty, and the decreased clinical and functional outcomes after partial and total meniscectomy for up to 40 years follow-up [9–12]. Consequently, many efforts have been made to develop meniscal substitutes in order to prevent early-onset knee OA, a condition that typically results in a partial- or total knee replacement [13–15].

The purpose of the present article is to provide a comprehensive review on the development, current state of research, clinical relevance, and future perspectives of meniscal substitutes.

## Anatomical and biomechanical considerations

Due to the unique composition and three-dimensional structure, the menisci are indispensable for physiological knee function. Key functions of the menisci include load-bearing, improvement of the femoro-tibial congruency, and, in combination with the meniscal ligament complex, a secondarily stabilizing effect. Further important roles are the contribution to joint lubrication, proprioception, and the distribution of intraarticular nutrients [16]. Given the relative movement between the menisci and the tibial plateau, the menisci form a dynamically acting joint socket which enhances the femoro-tibial congruency across the entire range-of-motion (ROM) [17, 18]. This dynamic behavior is especially important when considering the fixation technique of meniscal allografts in order to best restore the native knee mechanics and kinematics [19].

There are three distinct layers when describing the cross-sectional area of the meniscal tissue [20]. Thick bundles of circumferentially orientated collagen fibers are located in the central main layer, which are responsible for the mechanical properties of the menisci, especially for tensile strength. Additional fibers are radially orientated, or build a meshwork in the superficial and lamellar layer, contributing to the anisotropic behavior of the meniscal tissue [16, 20].

The sophisticated combination of composition, morphology, and anatomical attachments of the menisci, transforms the axially acting compression force during load-bearing into a circumferentially orientated tensile force (hoop-stress) [16]. As a result, 50% and 70% of the compartmental load is absorbed by the medial and lateral meniscus, respectively [21]. Following a total medial meniscectomy, the local peak contact pressure in the medial compartment increases by approximately 235%, highlighting the importance of the medial meniscus in load-bearing [22]. Similar results have been observed for the lateral compartment after total lateral meniscectomy [23]. Radial meniscal tears lead to a partial or total disruption of the circumferentially orientated collagen fibers. As a result, no hoop-stress can be transferred, which leads to pathological meniscal extrusion [24]. This condition causes an increase in the tibiofemoral contact pressure, which is why it is often termed „functional meniscectomy “[25–27]. Meniscal extrusion has gained interest in recent years and can be quantified by MRI or ultrasound [24, 28, 29]. It has been shown, that excessive meniscal extrusion is an indirect sign for radial tears as well as for meniscal root lesions [24, 29]. Radial meniscal displacement of > 3 mm is often considered as a cut-off value from physiologic to pathologic extrusion. However, a recent study has shown that meniscal extrusion can be a temporary and reversible observation after strenuous physical activity [28]. Therefore, the critical value for physiological meniscal extrusion continues to be the subject of current research.

**Fig. 1:**
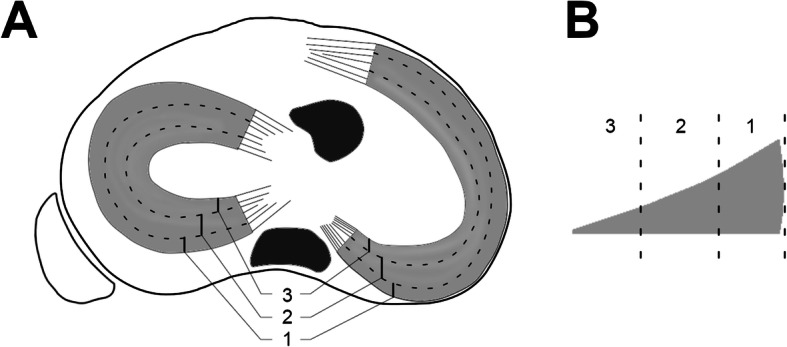
Meniscal zones according to [3, 31]. **a** Proximal view of the tibial plateau. **b** Cross-sectional area of a representative meniscus. The dotted lines divide the menisci into three different zones: 1, peripheral zone; 2, middle zone; 3, central zone

The vascular supply of the menisci is limited to the peripheral 10–25% by a perimeniscal capillary plexus [30]. This refers to zone 1 according to the proposed radial zones of the menisci (Fig. 1) [3, 31]. The more central zones 2 and 3 have less vascular supply. Therefore, the healing potential of meniscal tears is limited, especially for tears located in zone 3.

### Knee after total meniscectomy

Partial or total meniscectomy leads to an alteration of the biomechanics and kinematics of the knee [22, 32, 33]. Although meniscal resection may be followed by a rapid improvement of clinical symptoms, a subset of patients suffer from nagging pain after a certain period of time. This clinical presentation is termed “post-meniscectomy syndrome” and may be accompanied by radiological signs such as bone marrow edema [34]. Given the bony morphology of the tibiofemoral joint, it is important for the menisci to increase the tibiofemoral congruency. This is particularly true for the lateral compartment due to the convex lateral tibial plateau, which is reflected in the devastating effects following total or partial lateral meniscectomy [21, 35–38]. One study has shown that in patients undergoing total meniscectomy, the need for total knee arthroplasty is 132 times higher at 40-years follow-up due to symptomatic knee OA compared to an age-matched regional control population [11]. Non-surgical treatment for post-meniscectomy syndrome includes physical therapy, activity modification, and the use of non-steroidal anti-inflammatory drugs [34]. However, the evidence for non-surgical treatment remains limited and therefore surgical treatment options have to be considered, especially in young and active patients.

### Alignment and stability

Lower limb alignment essentially affects the load distribution across the medial and lateral compartment. Increasing varus malalignment and increasing loss of medial meniscal tissue (i.e. partial/total medial meniscectomy) lead to elevated tibiofemoral contact pressure in the medial compartment [39, 40]. Furthermore, a positive correlation between medial meniscal extrusion and tibiofemoral contact pressure exists [41]. This provides evidence that the medial meniscus is subjected to increased stress in varus malalignment. As previously mentioned, the menisci act as secondary restraints to anterior tibial and combined rotatory loads, becoming primary stabilizers in the anterior cruciate ligament (ACL) deficient knee [32]. In addition, ACL deficiency exposes the menisci to increased movement, deformation and stress [42, 43]. Clinically, chronic knee instability in patients with ACL deficiency increases the risk of medial meniscus injuries. Even a delay of 6 months in ACL reconstruction significantly increases the odds of sustaining a secondary medial meniscal lesion, which almost doubles after 12 months [44, 45].

Taken together, it is recommended to assess and, if necessary, address lower limb alignment and knee instability prior to or in combination with meniscal substitution to protect the meniscal substitute.

## Principles of meniscal substitution

Four main principles for the replacement of meniscal tissue exist. Total replacement can be performed with autogenous [46, 47] or allogenic transplants [48–51] as well as with artificial meniscus prostheses [52, 53]. For partial replacement, after extensive partial meniscectomy, artificial scaffold-based meniscal substitutes are available [54, 55].

Historically, total meniscal substitution was performed by using autografts like tendon, fat, or perichondral tissue. The autografts are intended to provide a three-dimensional scaffold, which facilitates the immigration of endogenous cells to stimulate a remodeling process. As a result, meniscus-like tissue is formed [56–58]. Similarly, total meniscal replacement by meniscal allograft transplantation (MAT) uses a human donor meniscus, which also provides a scaffold while simultaneously best replicating the morphological characteristics and biomechanical properties of the lost meniscal tissue [59]. Total medial meniscal replacement can also be performed by the use of artificial meniscus prostheses. Non-degradable, anatomically or non-anatomically shaped meniscus prostheses are inserted into the medial compartment to replace the lost meniscal tissue [52, 53]. To date, no evidence is available for lateral meniscal replacement by meniscus prostheses. Artificial scaffold-based meniscal substitutes, designed for partial meniscal replacement, provide an organically compounded three-dimensional scaffold which facilitates the ingrowth of cells to form new meniscus-like tissue. However, the initially implanted substitute is not designed to restore the biomechanical properties of the lost meniscal tissue. The ingrowth of fibrochondrocyte-like cells induces tissue formation and remodeling, which then resembles the biomechanical properties of the native meniscus [55, 60, 61].

### Autologous substitutes

Various types of autologous tissue for meniscal substitution have been reported. In one study, total medial meniscal replacement was performed with an ipsilateral mid-third patellar tendon autograft in a sheep model. After 12 months, tissue remodeling of the tendon autograft (tendon-meniscus) with incorporated chondrocyte-like cells could be observed. Despite morphological and histological similarities of the native meniscus and the tendon-meniscus, the biomechanical properties of the autograft were significantly worse compared to the native meniscus [58]. Similar morphological results but worse biomechanical properties were found when using a pediculated infrapatellar fat pad autograft in another sheep model [57]. A further attempt for total meniscal replacement was the use of autologous perichondral tissue harvested from the lower rib in a sheep model. Again, the autograft underwent remodeling but possessed inferior biomechanical properties compared to the native meniscus. Interestingly, the integrity of the articular surface was better preserved in the perichondral-meniscus group than in the meniscectomized group [56]. In spite of some promising results in animal studies, no favorable clinical results have been reported for autologous meniscal substitutes and therefore no routine clinical use has been established [46, 47].

### Allogenic substitutes

#### Meniscal allograft transplantation (MAT)

Meniscal allograft transplantation is the standard-of-care for the treatment of symptomatic post-meniscectomy syndrome after sub−/total meniscal resection. Indications and contraindications for MAT are listed in Table 2.

**Fig. 2:**
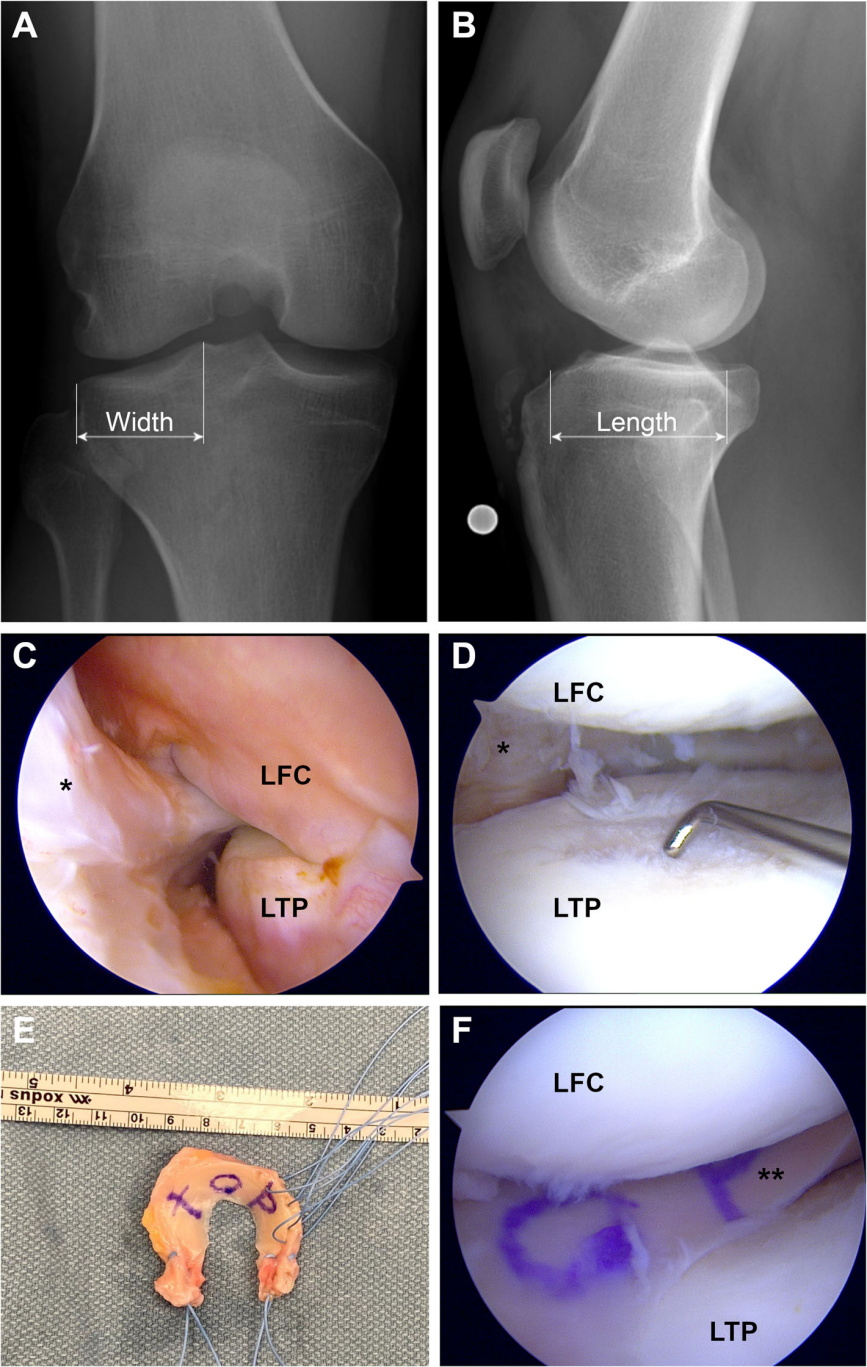
Lateral meniscal allograft transplantation (MAT) by suture-only fixation technique in a patient having undergone subtotal lateral meniscectomy without rim preservation (right knee). Preoperative **a)** anterior-posterior (AP) and **b)** lateral radiographs for sizing of the meniscal allograft. The width is measured on the AP radiograph as the distance from the lateral border of the tibial plateau to the apex of the lateral tubercle of the intercondylar eminence. The length is determined as 70% of the measured length of the lateral tibial plateau [63]. **c)** Arthroscopic view of the lateral gutter. Note the absence of meniscal tissue between the lateral femoral condyle (LFC) and the lateral tibial plateau (LTP); **d)** Arthroscopic view of the lateral compartment with grade 2 femoral and tibial cartilage lesions; **e)** Lateral meniscal allograft after preparation and application of the fixation sutures; **f)** Arthroscopic view of the lateral compartment after lateral MAT; *,Remnant of the native lateral meniscus; **, Lateral meniscal allograft in situ

After a thorough arthroscopic debridement of the affected compartment, a size-matched meniscal allograft is inserted into the knee through one of the arthroscopic portals or a mini-open arthrotomy. In principle there are two different techniques for intraarticular graft fixation: with bone block and suture-only. For the bone block fixation technique, a distinction between medial and lateral MAT has to be made. For lateral MAT in the bone block technique, a tibial trough, which connects the anterior and posterior meniscal roots, is created. For press-fit fixation, the bone block, which is attached to the meniscal allograft roots, is pushed into the trough [19]. In contrast, for medial MAT, two small bone plugs, which are attached to the anterior and posterior root of the medial meniscal allograft, are inserted into the respective tibial bone tunnel and fixed in a transtibial pull-out fashion [62]. Additional soft tissue fixation is performed utilizing inside-out, outside-in or all-inside meniscal sutures, as appropriate. For the suture-only fixation technique (Fig. 2), the meniscal allograft roots are fixed by two transtibial pull-out sutures. Additional fixation of the meniscal rim is performed by the use of inside-out, outside-in or all-inside meniscal sutures, as appropriate [19, 48–50, 59]. For rehabilitation, many different approaches are described after MAT. Mostly, ROM exercises are started within the first postoperative week to achieve full ROM after 8 weeks. With regard to weight-bearing, 4 weeks and 6 weeks are recommended to start partial and full weight-bearing, respectively [64].

As MAT has substantially evolved since the 1980s, clinical data with emphasis on the different fixation techniques, medial vs. lateral MAT, return-to-play, failure and re-operation rates as well as long-term outcomes of many trials have been published. Overall, patient reported outcomes (PROs) improve significantly after MAT from pre- to postoperative, both for medial and lateral MAT as well as for the bone block and the suture-only fixation technique [48–50, 59]. However, the superiority of either of the two fixation techniques remains unknown. In a recent matched-pair analysis, no difference between the bone block (*n* = 37) and the suture-only (*n* = 45) fixation technique could be observed regarding PROs and meniscal allograft extrusion measured by MRI [49]. In contrast, more favorable results are described for lateral MAT than for medial MAT. One meta-analysis comparing medial vs. lateral MAT showed that lateral MAT leads to higher Lysholm scores and improved pain relief compared to medial MAT at mid-term (5–10 years) and long-term (> 10 years) follow-up (FU) [65]. Encouraging clinical outcomes after MAT are also reflected in the high return-to-play rate. A recent systematic review reported a return-to-play rate of 77% (483/624) after MAT. The average reported time from surgery to return-to-play is 9 months and close to 70% (326/475) return to the same or a higher sports level [64]. However, the main goal of MAT is pain relief and a subsequent improved quality of life. This has to be discussed with every patient preoperatively to avoid unrealistic expectations. Accordingly, patients should be counseled against a return to high level sports. Although the weighted average satisfaction rate after MAT is 82%, the respective complication rate is 11% [59]. One study demonstrated a re-operation rate of 32% after MAT (172 patients, 59 months mean FU) [51]. Tearing of the meniscal allograft is described to be the most common complication [59] and a simple arthroscopic debridement the most frequently performed re-operation procedure [51]. However, the survival rate of MAT is reported to be 86% (medial) / 89% (lateral) at mid-term (5–10 years) and 52.6% (medial) / 57% (lateral) at long-term (> 10 years) [65].

Taken together, MAT represents the standard-of-care in the treatment of patients with symptomatic post-meniscectomy syndrome. Yet this procedure has some inherent limitations. First, the meniscal allograft has to fit properly in size in order to achieve its desired benefits. Oversizing of the meniscal allograft results in increased tibiofemoral contact pressure compared to the native condition, since the oversized graft is not capable of transforming compressive load into hoop-stress and thus limits the ability of load transmission [66]. On the other hand, undersizing increases the stress within the meniscus, leading to a higher risk of graft failure [66]. Second, meniscal allografts are not readily available in many countries, which limits their broad clinical use.

### Artificial substitutes

#### Scaffolds

Two artificial, scaffold-based, and biocompatible meniscal substitutes for partial meniscal replacement are commercially available for clinical use. The collagen meniscus implant (CMI; Stryker Corporation, Kalamazoo, MI, USA), which consists of type-I collagen fibers derived from bovine Achilles tendons, has gained attention since the first clinical trial was published in 1997 [54]. The second, more recently developed artificial meniscal substitute is labelled Actifit® (Orteq Sports Medicine Ltd., London, United Kingdom), which represents a synthetic hybrid of polycaprolactone (80%) and polyurethane (20%) and was first described in a clinical trial in 2011 [55]. A detailed synopsis of published clinical studies on scaffold-based meniscal substitutes is shown in Table 1.

**Table 1:** Synopsis of clinical studies on scaffold-based meniscal substitutes (chronological order)

Author	Year	N	Age^|^	♂ / ♀	FU	Scaffold	M/L	A/C	Size^||^	Assoc. Procedures	Radiologic Outcomes	Clinical Outcomes
			[years]		[months]				[mm]	[%]		
Toanen [67]	2020	155	34	109/46	60	Actifit®	101/54	14/141	39.4	HTO (28), ACLR (19), CS (4)	Genovese score, extrusion, ICRS	VAS, LS, IKDC, KOOS, survival rate
Schenk [68]	2019	39	34	30/9	36	CMI	32/7	25/14	48	ACLR (62)	Genovese score, extrusion	VAS, LS, IKDC, Tegner, clinical failure
Monllau [69]	2018	32	41	25/7	70.8	Actifit®	21/11	N/A	40.9	ACLR (28), HTO (41), PCLR (3), CS (44)	Genovese score, extrusion, volume	LS, IKDC, KOOS, Tegner,
Leroy [70]	2017	15	30	8/7	72	Actifit®	6/9	0/15	N/A	ACLR (33), CS (7)	Genovese score, extrusion, ICRS	VAS, IKDC, KOOS, failure rate
Dhollander [71]	2016	44	32	24/20	60	Actifit®	29/15	4/40	45.5	ACLR (9), HTO (9)	Genovese score, extrusion, ICRS	VAS, IKDC, KOOS, survival rate
Filardo [72]	2016	16	45	9/7	72	Actifit®	12/4	N/A	N/A	ACLR (38), HTO (13), ME (94), CS (13), O (6)	Genovese score, extrusion	IKDC, Tegner
Schüttler [73]	2015	18	33	N/A	48	Actifit®	18/0	0/18	44.5	None	Genovese score, extrusion	VAS, KOOS, KSS, UCLA
Faivre [74]	2015	20	29	N/A	24	Actifit®	8/12	0/20	N/A	Ligament reconstruction (20), CS (15)	Signal intensity, extrusion, cartilage coverage index	IKDC, KOOS
Martín-Hernández [75]	2015	10	31	4/6	24	Actifit®	9/1	0/10	N/A	None	Genovese score, extrusion	VAS, LS, KOOS
Schüttler [76]	2015	18	33	N/A	24	Actifit®	18/0	0/18	40.9	None	Genovese score, extrusion	VAS, KOOS, KSS, UCLA
Baynat [77]	2014	18	20–46	13/5	24	Actifit®	13/5	0/18	N/A	ACL (56), HTO (56)	Genovese score, extrusion	LS, Histology
Gelber [78]	2014	30	51	40/20	31.2	Actifit®	60/0	0/60	40.3	HTO (100)	Radiographic evaluation of limb alignment and tibial slope	VAS, IKDC, Kujala, WOMET,
Bouyarmane [79]	2014	54	28	37/17	24	Actifit®	0/54	0/54	43	ACLR (17), DFO (9)	N/A	VAS, IKDC, KOOS
Kon [80]	2014	18	45	11/7	24	Actifit®	13/5	1/17	N/A	ACLR (17), Osteotomy (22), CS (39), O (17)	Genovese score	IKDC, Tegner
De Coninck [81]	2013	26	35	12/12	24	Actifit®	18/8	N/A	43.2	ACLR (23), HTO (4), DFO (4), CS (4), MAT (4)	Meniscal rim thickness, extrusion	VAS, LS, IKDC, KOOS
Bulgheroni [82]	2013	20	33	17/2	24–46	Actifit®	17/3	0/20	43/37**	ACLR (47), HTO (37), DFO (5)	Genovese score	VAS, LS, Tegner, second-look
Hirschmann [83]	2013	67	36	47/20	12	CMI	55/12	42/25	N/A	ACLR (67), HTO (7), CS (4)	Genovese score, extrusion	VAS, LS, IKDC, Tegner, clinical failure
Zaffagnini [84]	2012	24	36	20/4	26	CMI	0/24	7/17	45.2	ACLR (17), CS (25), O (4)	Genovese score, Yulish score	VAS, LS, IKDC, Tegner, EQ-5D
Efe [85]	2012	10	29	8/2	12	Actifit®	10/0	0/10	39.2	None	Genovese score, bone bruise, ICRS, remaining meniscus	VAS, KOOS, KSS, UCLA
Verdonk [86]	2012	52	31	39/13	24	Actifit®	34/18	2/46 (4 unknown)	47.1	ACLR (4)	ICRS	VAS, LS, IKDC, KOOS, SAE
Verdonk [55]	2011	52	31	39/13	12	Actifit®	34/18	2/46 (4 unknown)	47.1	N/A	Tissue ingrowth (DCE-MRI), ICRS	Pain, functionality, quality of life, Histology
Zaffagnini	2011	17	40	33/0	133	CMI	33/0	17/16	36	ACLR (12)	Genovese score, Yulish score, Radiographic evaluation	VAS, LS, IKDC, Tegner, SF-36
Monllau [87]	2011	25	29	20/5	133.2	CMI	25/0	20/5	48.2	ACLR (56), CS (4)	Genovese score, radiographic progression of OA	VAS, LS, failure rate,
Bulgheroni [88]	2010	34	39	25/9	60	CMI	34/0	6/28	45	ACLR (32), HTO (6), CS (3)	Genovese score, Yulish score, radiographic progression of OA	LS, Tegner, Histology
Rodkey [61]	2008	160	39	243/68	59	CMI	311/0	157/154	N/A	ACLR (27)	N/A	VAS, LS, Tegner, Histology
Linke [89]	2007	23	42	N/A	24	CMI	23/0	N/A	N/A	HTO (100)	N/A	VAS, LS, IKDC
Genovese [90]	2007	40	41	27/13	12–24	CMI	40/0	28/12	N/A	ACLR (40), HTO (3), CS (4)	Genovese score	N/A
Steadman [91]	2005	8	40	8/0	69.6	CMI	8/0	1/8	N/A	None	Signal intensity, interface, cartilage, bone marrow, radiographic evaluation of OA	VAS, LS, IKDC, Tegner, Histology
Rodkey [92]	1999	8	40	N/A	24	CMI	8/0	1/7	42.5	None	Morphology, size, radiographic progression of OA	VAS, LS, Tegner, Histology
Stone [54]	1997	10	39	8/2	36	CMI	10/0	4/6	N/A	ACLR (20)	Signal intensity, interface, radiographic evaluation of OA	VAS, activity + performance score, overall knee rating, Histology

Abbreviations: *A/C* Acute/chronic; *ACLR* Anterior cruciate ligament reconstruction; *CMI* Collagen meniscus implant; *CS* Cartilage surgery; *DCE-MRI* Dynamic contrast-enhanced magnetic resonance imaging; *DFO* Distal femoral osteotomy; *EQ-5D* EuroQuol 5 dimensions; *FU* Follow-up; *HTO* High tibial osteotomy; *ICRS* International cartilage repair society; *IKDC* International knee documentation committee; *KOOS* Knee injury and osteoarthritis outcome score; *KSS* Knee society score; *LS* Lysholm score; *M/L*, medial/lateral; *MAT* Meniscal allograft transplantation; *ME* Meniscectomy; *N* Number of patients treated *with* scaffold-based meniscal substitutes; *N/A* Not available; *O* Others; *OA* Osteoarthritis; PCLR Posterior cruciate ligament reconstruction; *SAE* Scaffold-related serious adverse events; *SF-36* Short form 36; *UCLA* University of California, Los Angeles activity scale; *VAS* Visual analogue scale; *WOMET* Western Ontario meniscal evaluation tool; ^|^,mean age at surgery; ^||^, mean defect size

**Fig. 3:**
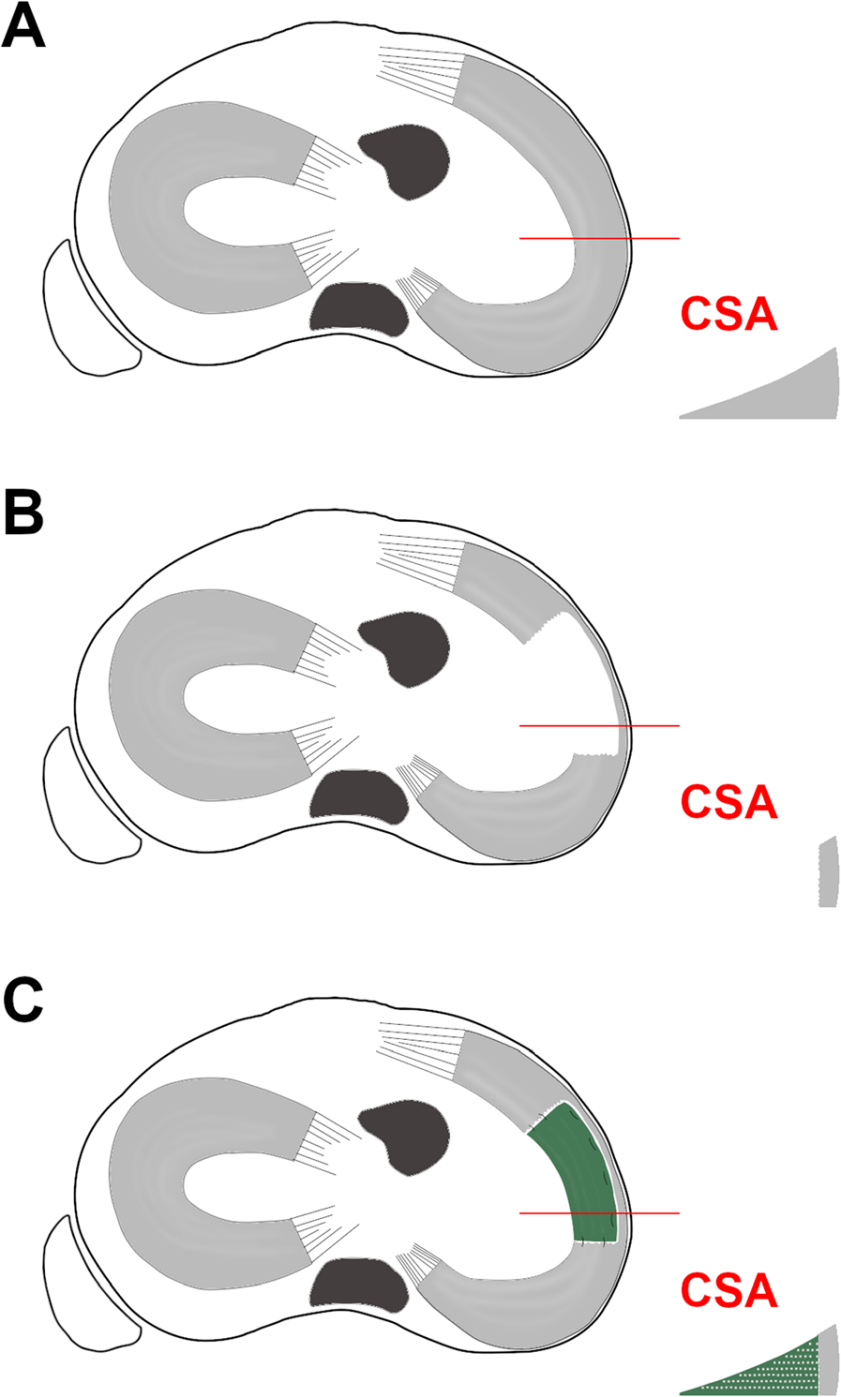
Schematic illustration of scaffold-based partial medial meniscal replacement. Proximal view of the tibial plateau and the respective cross-sectional area (CSA) of the medial meniscus at the level of the red line for the following conditions, **a)** Intact; **b)** After extensive partial medial meniscectomy; **c)** After scaffold-based partial medial meniscal replacement. Note the framework of the meniscal substitute

**Fig. 4:**
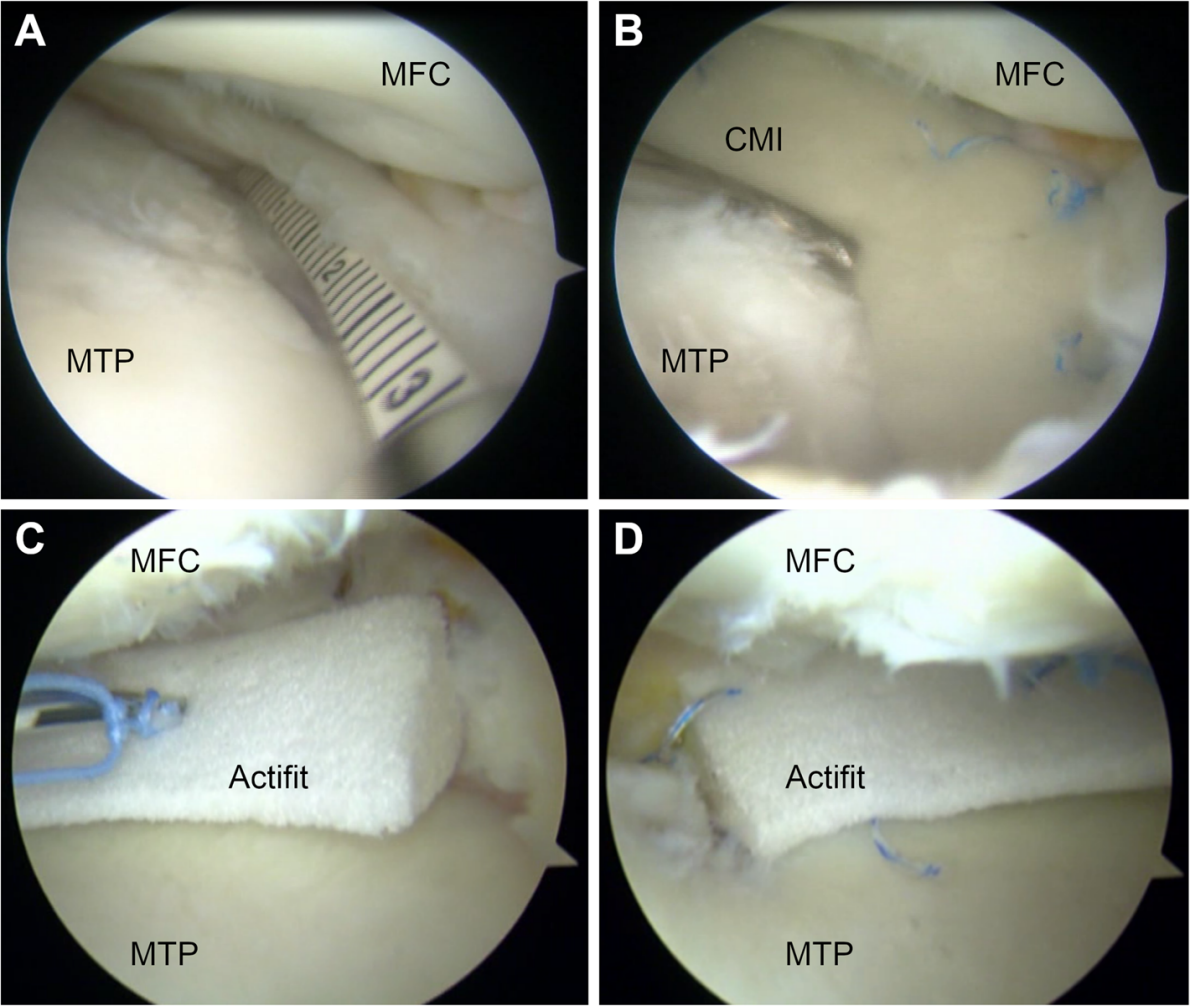
Arthroscopic images of scaffold-based partial meniscal substitution. **a)** and **b)** Substitution of partial medial meniscus defect using the collagen meniscus implant (CMI; Stryker Corporation, Kalamazoo, MI, USA). **c)** and **d)** Substitution of partial medial meniscus defect using Actifit® (Orteq Sports Medicine Ltd., London, United Kingdom). **a)** Measurement of the defect size. **b)** CMI in situ. **c)** All-inside suture fixation of Actifit®. **d)** Actifit® in situ. MFC, medial femoral condyle; MTP, medial tibial plateau. (Acknowledgement to Stefano Zaffagnini, who provided the images)

Once the indication (Table 2) for partial meniscal substitution has been proven and a thorough debridement of the partially meniscectomized area has been performed, the size of the scaffold is determined by an arthroscopic ruler. Thereafter, the implantation of the slightly oversized (+ 10% of the debrided area) scaffold is performed arthroscopically. All-inside, inside-out, or outside-in suture techniques are used for substitute fixation to the surrounding meniscal tissue, as appropriate [55, 61, 92]. A schematic illustration and clinical examples of scaffold-based partial meniscal substitution are shown in Fig. 3 and Fig. 4, respectively. For postoperative rehabilitation, a hinged knee brace is recommended. Continuous passive ROM exercises limited to 60° and 90° of knee flexion for 3 to 6 weeks postoperatively, respectively, should be performed. Partial weight-bearing in full knee extension is allowed after 3 weeks, followed by free active ROM and full weight-bearing after 6 to 9 weeks [55, 61].

**Table 2:** Indications and contraindications for scaffold-based meniscal substitutes and meniscal allograft transplantation

	Scaffold-based meniscal substitution	Meniscal allograft transplantation
**Indications**	Clinical symptoms s/p extensive partial meniscal resection Stable meniscal rim Intact meniscal roots Chronic partial meniscal defect	Clinical symptoms s/p sub−/total meniscal resection Insufficient meniscal rim Insufficient meniscal roots
**Contraindications**	Age: > 50 years^a^ BMI: > 35^a^ Insufficient meniscal rim Insufficient meniscal roots Knee instability Limb malalignment Allergies to animal derived products Meniscal defect limited to zone 3 (Fig. 1) ICRS grade > 3 Active infection Autoimmune diseases Inflammatory arthritis Smoker^a^	Age: > 50 years^a^ BMI: > 35^a^ Outerbridge grade III, IV Fairbank grade > 2 Joint space narrowing Knee instability Limb malalignment Active infection Autoimmune diseases Inflammatory arthritis Smoker^a^

Abbreviations: *ICRS* International cartilage repair society; *s/p*, status post; ^a^, relative contraindication

Almost every clinical trial investigating clinical outcomes after scaffold-based partial meniscal replacement reports improvements of PROs at follow up (Table 1). However, comparative studies are scarce [61, 78, 93]. A randomized, controlled, multicenter clinical trial showed that patients treated with CMI regained significantly higher activity (Tegner Activity Scale) and were more satisfied compared to patients treated with partial medial meniscectomy. No difference could be observed between the group with irreparable medial meniscal tears and acute replacement with CMI and the group treated with partial medial meniscectomy [61]. Similar results were shown by a randomized controlled trial with a minimum of 10 years follow up [93]. A recently published multicenter study showed an improvement in PROs after Actifit® implantation at 2- and 5-years FU compared to the preoperative condition. Clinical outcome data are reported for 137 patients and show consistency between 2 and 5 years postoperatively. No difference could be demonstrated between medial and lateral implantation with a mean overall survival rate of the meniscal scaffold of 92% and 88% at 2- and 5-years FU, respectively [67]. However, clinical failure, defined as scaffold-related infections, mechanical failure, chronic synovitis or the need for re-operation is reported in up to 8% and 32% for the CMI and Actifit®, respectively [67, 70, 71, 83, 87].

Since knee joint instability and lower limb malalignment are contraindications for scaffold-based partial meniscal substitution, concurrent interventions are frequently performed, with ACL reconstruction, high tibial osteotomy, and cartilage procedures being the most common [67, 72, 77, 78, 89]. A recent study showed that a combined high tibial valgus osteotomy and medial meniscus scaffold-based partial substitution does not lead to better clinical outcomes – based on PROs – than a combined high tibial osteotomy with partial medial meniscus resection. Therefore, the authors did not recommend the combination of high tibial valgus osteotomy and partial medial meniscal substitution, as lower limb realignment appeared to achieve the most important clinical improvement [78].

The clinical benefit of scaffold-based partial meniscal substitution is more evident in the chronic rather than acute settings (Table 1). This appears to be controversial, since a loss of meniscal tissue has been shown to instantly alter knee joint biomechanics [32, 33]. The rapid improvement of clinical symptoms after partial meniscectomy following an acute meniscal lesion may be an explanation. In contrast, the process of remodeling and adjustment to an implanted meniscal scaffold requires more time, but might be favorable regarding long-term outcomes [94], since tissue ingrowth within the scaffold and formation of meniscus-like tissue is evident according to histological analysis and second-look arthroscopy [54, 55, 61, 77, 88, 91, 92]. Consequently, the devastating effects of partial meniscectomy may be prevented and the development of knee OA delayed [9, 10, 12].

#### Meniscus prostheses

Anatomically [53, 95] and non-anatomically [52, 96] shaped artificial meniscal substitutes (prostheses) made of polycarbonate-urethane represent novel techniques for total meniscal replacement. In spite of intensive research efforts, experiences to date are limited.

In one study, the condition of an anatomically shaped total meniscal substitute and its chondroprotective effect was investigated with 12 months FU in a goat model. Although the integrity of the total meniscal substitute, in terms of wear and deformation, was maintained, no difference in the cartilage histopathological degeneration could be observed between the groups of total meniscal substitution, meniscal allograft transplantation and total meniscectomy. Additionally, the total meniscal substitute was not chondroprotective compared to a non-operative control group [53]. A biomechanical study on human cadaveric specimens showed that the same anatomically shaped total meniscal substitute could not restore native contact mechanics and knee kinematics. Based on the results of this study, there is no significant difference between total meniscal substitution and MAT [95].

NUsurface® Meniscus Implant (Active Implants LLC, Memphis, TN, USA) represents a non-anatomically discoid-shaped, free floating and non-anchored meniscal substitute designed for total replacement of the medial meniscus [52, 96]. A biomechanical study showed that implantation of the NUsurface® Meniscus Implant restores the average and peak tibiofemoral contact pressure to 93% and 92%, respectively, compared to the native medial meniscus [96]. In a case-series of three prospectively enrolled patients, no differences for knee and meniscal kinematics between knees with implanted medial NUsurface® Meniscus Implant and the contralateral healthy knees could be observed based on MRI studies. However, clinical outcome measures are not mentioned in this study [52]. Although the clinical use of the NUsurface® Meniscus Implant started in Europe and Israel in 2008 and 2011, respectively, evidence-based clinical data remains largely absent. Two clinical trials are currently ongoing in the USA, with FDA approval for the NUsurface® Meniscus Implant still pending [97].

## Clinical indication

Generally, the complaints and clinical symptoms of the patient are the primary predictors in treatment decision-making [98]. The ideal patient for scaffold-based meniscal substitution has a history of extensive partial meniscectomy with the peripheral meniscal rim remaining intact, pain localized to the respective joint line, full range of motion, has no signs of knee instability, no malalignment, or high-grade chondral defects, and has failed non-operative treatment [61, 67, 93]. On the other hand, an insufficient meniscal rim and a previously performed sub−/total meniscectomy represent contraindications for partial meniscal substitution and MAT is the standard-of-care in such cases [59]. Due to poor clinical data, autologous meniscal substitutes have not gained acceptance for clinical practice. Total meniscal replacement on the basis of meniscus prostheses has recently been established for clinical use. However, comprehensive clinical data are still pending.

The indications and contraindications for scaffold-based meniscal substitution and MAT are listed in Table 2.

## Tissue engineering and future perspectives

Tissue engineering, under the broader umbrella of regenerative medicine, entails the independent or combined application of cells, bioactive agents (e.g., growth factors, viral vectors, small bioactive peptides), scaffolds, and biophysical stimuli, to fabricate biological substitutes that restore, maintain, or improve tissue function [99]. As noted above, clinically available meniscal substitutes, including non-meniscus autografts (e.g., tendon, adipose), MAT, meniscal prostheses, and scaffolds (i.e., CMI and Actifit®), each possess unique advantages and disadvantages. None restore the exact morphology, biochemical composition, ultrastructure, and cellular phenotypes of the native meniscus, let alone matching these properties for each unique patient. Meniscal tissue engineering ultimately seeks to overcome the limitations of current meniscal substitutes by fabricating a scaffold that is either seeded with cells prior to implantation or sequentially recruits and orchestrates meniscus-specific differentiation of endogenous progenitor cells, thereby serving as a patient-specific autograft.

Decellularized extracellular matrix (dECM) derived from allograft or xenogeneic meniscus largely possesses the ultrastructural, biochemical composition, and bioactive motifs of native meniscus with elimination of the immunogenic foreign cells [100, 101]. The preserved microenvironment of the native tissue promotes meniscus- and even region-specific cellular differentiation by dECM [102, 103]. Preservation of bioactive motifs is possible with further processing of the meniscus dECM into powders, hydrogels, and water-soluble protein cocktails, which may impart meniscus-specific bioactivity when combined with other biomaterials [102, 104]. For instance, a 3-dimensional- (3D-) printed polycaprolactone (PCL) scaffold in the shape of the meniscus was augmented with meniscus dECM hydrogel [105]. When seeded with meniscal fibrochondrocytes (MFCs), the hybrid-scaffolds enhanced cell proliferation, chondrogenic differentiation, glycosaminoglycan and collagen production, and mechanical properties, as compared to the PCL scaffold without dECM [105]. Six months following implantation as a meniscal replacement into a rabbit model of total medial meniscectomy, the PCL-dECM-MFC construct had similar histological structures, biochemical contents, and biomechanical properties as the native meniscus, a result that was superior to both a PCL scaffold and PLC-dECM construct [105].

While meniscus dECM has shown promising results in both in vitro and preclinical models, its procurement is labor-intensive and prone to batch-to-batch variability, with additional limitations in size matching for total meniscal replacement. The combined use of defined biodegradable polymers and bioactive factors (e.g., growth factors) affords greater control over the fabrication process of engineered scaffolds. Comprehensive reviews of the myriad materials and methods employed to fabricate tissue-engineered scaffolds have been published [106, 107]. As an example, a PCL scaffold was 3D-printed through computer-assisted design (CAD) modeling of a 3D laser scan of the native meniscus [108]. Using growth-factor impregnated microspheres of biodegradable polymers, the controlled-release of transforming growth factor beta 3 (TGF- β3) and connective tissue growth factor (CTGF) was respectively localized to the inner and outer regions of the scaffold, providing region-specific chondrogenic and fibrochondrogenic differentiation of seeded mesenchymal stem cells (MSCs), as tested in vitro [108]. When implanted in an ovine model of medial meniscal replacement, the acellular scaffold with zone-specific TGF-β3 (inner) and CTGF (outer) microspheres recruited endogenous progenitor cells, promoted type II collagen deposition in the inner region and type I collagen deposition in the outer region that was reminiscent of the native meniscus, and restored inhomogeneous mechanical properties [108]. These promising results were seen at 3 months following implantation. Unfortunately, when animals were followed up to 1 year, there was cartilage degeneration observed grossly and meniscal extrusion evident on MRI in most animals, suggesting that the engineered scaffold did not perfectly restore native meniscal function [109]. As for all tissue-engineered scaffolds, long-term studies examining the potential chondroprotective effect of the biomaterial are needed but infrequently performed. As with existing meniscal substitutes, controlling degradation/remodeling rates and preventing meniscal extrusion of engineered scaffolds remain challenges for optimizing the benefit of these promising biomaterials.

**Fig. 5:**
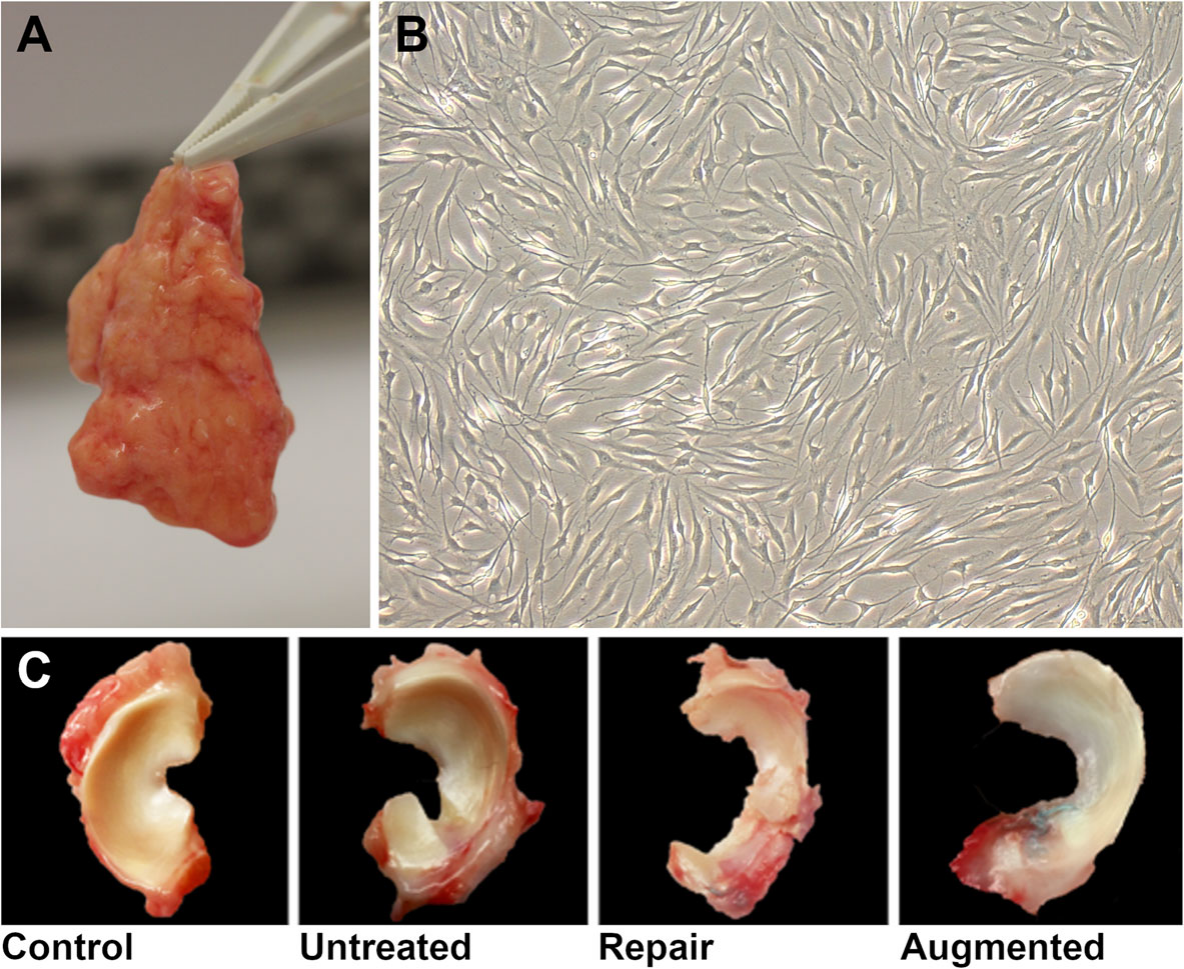
Augmented meniscal repair in a goat model. **a)** Isolated infrapatellar fat pad (IPFP) **b)** IPFP-derived mesenchymal stromal cells under microscope demonstrating characteristic spindle-shaped morphology **c)** Goat meniscus 6 months after creation of a critical-sized radial meniscal tear in 4 different groups: Control, no meniscal tear; Untreated, radial meniscal tear left in situ; Repair, radial tear repair with single horizontal mattress suture; Augmented, radial tear repair with single horizontal mattress suture + augmentation with cell-seeded hydrogel at the tear site. Suture repair minimized gap formation and neotissue formation was further improved with cell-based augmentation

Meniscal replacement is the most ambitious application of tissue engineering, but the strategies may also be applied to promote localized meniscus regeneration, as for instance, to augment suture repair techniques. Intraarticular injection of MSCs into animal models of meniscus tears and/or partial meniscectomy have been found to improve healing and promote regeneration as determined by macroscopic observation, histological scoring, and MRI imaging [110–112]. More commonly, MSCs (or fibrochondrocytes/chondrocytes) seeded in a cell carrier (e.g., hydrogel, scaffold) are localized to the site of damage. Not only does the cell carrier concentrate the MSCs and their pro-healing paracrine factors at the tear site, but it can serve to concurrently release bioactive factors and/or provide structural support to the cells and resulting neotissue. In two related studies, injection of MSCs encapsulated in a thermosensitive [113] or photocrosslinkable [114] hydrogel into a meniscal tear in a rabbit or goat repair model, respectively, enhanced healing especially when the hydrogels where concomitantly supplemented with pro-chondrogenic TGF-β isoforms. In the study using a goat model, the MSCs were rapidly isolated by enzymatic digestion of the infrapatellar fat pad, permitting a point-of-care (i.e., single stage) cell-based therapy for augmented meniscus repair [114] (Fig. 5).

Emerging technologies, including biomaterials, gene editing, cellular engineering, advanced imaging, and additive manufacturing (e.g. 3D printing), will provide expanding opportunity to repair, regenerate, or replace the injured meniscus. However, very few clinical studies have been performed at present. In the only prospective, randomized clinical study on MSC therapy for meniscus regeneration, 55 patients at 7 institutions who underwent partial medial meniscectomy were injected with one of three treatments – Group A: 50 × 106 allogeneic MSCs, Group 2: 150 × 106 allogeneic MSCs, or Group 3 (controls): sodium hyaluronate [115]. As determined by quantitative MRI, there was a significant increase in meniscal volume (defined a priori as 15% total meniscal volume) in 24% of patients in Group A, 6% of patients in Group B, and no patient in Group C. For those with osteoarthritic changes, both MSC groups reduced pain, as determined by a visual analog scale [115]. Several related case reports and case series with small sample sizes have been reported with generally positive findings, but absent the inclusion of comparative groups (preferably placebo controls), conclusions on the putative clinical benefit of tissue engineering strategies addressing meniscus injury remain limited [116].

Implementation of meniscal tissue engineering strategies into clinical practice must address both technical and regulatory challenges. Understanding the mechanisms by which cell-based therapies, and tissue engineering constructs more broadly, exert their effect has been obscured in part by imprecise nomenclature and the inconsistent and insufficient reporting of details of both the biological product and the resulting outcome measures [117]. Several professional societies with interest in advancing cell-based therapies and tissue engineering have issued guidelines to address these past limitations [118–120]. Further limiting more rigorous clinical investigation on the topic is the regulatory landscape set forth by governing health agencies. Notably, the United States’ Federal Drug Agency (FDA), which oversees the testing, approval, and use of cell and tissue products, categorizes nearly all tissue engineering constructs as “human cells, tissues, and cellular and tissue-based products (HCT/Ps)”, which require formal clinical trials to evaluate safety and efficacy [121]. Regulation is unequivocally needed to fulfill the FDA’s mission of protecting public health, but the current regulation of tissue engineering products, including adults MSCs, creates financial barriers that have heretofore often been prohibitive for researchers, companies, and investors, to pursue more widespread clinical testing. Recognizing the promise in tissue engineering, the FDA developed a comprehensive regenerative medicine policy framework in 2017, as regulatory bodies increasingly engage clinicians, researchers, and companies, to spur innovation while ensuring safety and efficacy. Engagement of all parties will be needed to bring transformative technologies to fruition, thereby improving the care of patients with challenging meniscus injuries.

## Conclusion

Remarkable research efforts over the past decades have enhanced the knowledge about the importance of the menisci for the knee, which has led to the development of meniscus-preserving and meniscus-replacing treatment options. While autologous meniscal substitution has not been successful, the techniques of meniscal allograft transplantation evolved to become the current standard-of-care for total meniscal insufficiency. Increasing clinical evidence for the efficacy of artificial scaffold-based meniscal substitutes has emerged for the treatment of irreparable partial meniscal injuries. The feasibility of cell-seeded scaffolds, the preservation of the meniscal microenvironment to promote cell proliferation and differentiation, and the development of materials that mimic the mechanical and viscoelastic properties of the native menisci are owed to the progress in tissue engineering. Therefore, the future of meniscal substitution is encouraging with the goal of ultimately improving outcome for patients with post-meniscectomy syndrome.

## Data Availability

Not applicable.

## Abbreviations

3D: 3-Dimensional
ACL: Anterior cruciate ligament
CMI: Collagen meniscus implant
CSA: Cross-sectional area
CTGF: Connective tissue growth factor
dECM: Decellularized extracellular matrix
FDA: Federal drug agency
FU: Follow-up
HCT/Ps: Human cells, tissues, and cellular and tissue-based products
MAT: Meniscal allograft transplantation
MFCs: Meniscal fibrochondrocytes
MSCs: Mesenchymal stem cells
OA: Osteoarthritis
PCL: Polycaprolactone
PROs: Patient reported outcomes
ROM: Range-of-motion
TGF- β3: Transforming growth factor beta 3
